# Insulin as a Bridge between Type 2 Diabetes and Alzheimer Disease – How Anti-Diabetics Could be a Solution for Dementia

**DOI:** 10.3389/fendo.2014.00110

**Published:** 2014-07-08

**Authors:** Inês Sebastião, Emanuel Candeias, Maria S. Santos, Catarina R. de Oliveira, Paula I. Moreira, Ana I. Duarte

**Affiliations:** ^1^Center for Neuroscience and Cell Biology (CNC), University of Coimbra, Coimbra, Portugal; ^2^Department of Life Sciences, University of Coimbra, Coimbra, Portugal; ^3^Institute for Interdisciplinary Research (IIIUC), University of Coimbra, Coimbra, Portugal; ^4^Institute of Biochemistry, Faculty of Medicine, University of Coimbra, Coimbra, Portugal; ^5^Institute of Physiology, Faculty of Medicine, University of Coimbra, Coimbra, Portugal

**Keywords:** Alzheimer disease, anti-type 2 diabetes compounds, brain, exendin-4, incretins/glucagon-like peptide-1/glucagon-like peptide-1 receptor, insulin/insulin receptor signaling, type 2 diabetes

## Abstract

Type 2 diabetes (T2D) and Alzheimer disease (AD) are two major health issues nowadays. T2D is an ever increasing epidemic, affecting millions of elderly people worldwide, with major repercussions in the patients’ daily life. This is mostly due to its chronic complications that may affect brain and constitutes a risk factor for AD. T2D principal hallmark is insulin resistance which also occurs in AD, rendering both pathologies more than mere unrelated diseases. This hypothesis has been reinforced in the recent years, with a high number of studies highlighting the existence of several common molecular links. As such, it is not surprising that AD has been considered as the “type 3 diabetes” or a “brain-specific T2D,” supporting the idea that a beneficial therapeutic strategy against T2D might be also beneficial against AD. Herewith, we aim to review some of the recent developments on the common features between T2D and AD, namely on insulin signaling and its participation in the regulation of amyloid β (Aβ) plaque and neurofibrillary tangle formation (the two major neuropathological hallmarks of AD). We also critically analyze the promising field that some anti-T2D drugs may protect against dementia and AD, with a special emphasis on the novel incretin/glucagon-like peptide-1 receptor agonists.

## Introduction

Given its ever increasing number of patients worldwide, diabetes mellitus (DM) is a modern epidemic and a highly concerning global health care issue, with recent estimates pointing to an increase from 285 to 439 million patients from 2010 to 2030 (1). Despite the several types of diabetes existent, type 2 diabetes (T2D) constitutes an unquestionable major public health threat, accounting for more than 90% of all cases (1, 2). T2D-related socio-economic concern is also due to the burden constituted by its highly common, morbid, and mortal long-term complications affecting several tissues and organs, including the central nervous system (CNS) (3). Although traditionally, T2D used to occur in people aged above 30 years, nowadays its incidence is also increasing among younger people (4), probably due to other risk factors (besides aging) associated with modern lifestyle. These include high blood pressure, sedentarism, and metabolic syndrome, the later constituting a group of disorders (e.g., dyslipidemia and obesity) related to a high risk for cardiovascular disease (1, 5, 6).

Type 2 diabetes is characterized by abnormally high blood glucose levels (hyperglycemia) due to insulin resistance that may progress toward pancreatic β-cell dysfunction and a generalized loss of insulin sensitivity in the later stages of disease (7). Chronic T2D is known to negatively affect the CNS structure and function, ultimately constituting a known risk factor for dementia [both vascular dementia and Alzheimer disease (AD)] (8). Although the precise mechanisms underlying neurodegeneration and impaired cognition in T2D remain poorly understood, it has been hypothesized that insulin resistance (impaired insulin signaling) may play a critical role, particularly in the elderly (9, 10). This is not surprising, giving that aging is a well-known common risk factor to both T2D and AD (11) and recent hypotheses suggest that AD might be a neuroendocrine-like disorder, a so-called “type 3 diabetes” (12, 13) or a “brain-specific T2D” (14). Accordingly, Janson et al. reported that both T2D and impaired fasting glucose were more prevalent in AD than in non-AD individuals from the Mayo Clinic Alzheimer Disease Patient Registry, with 81% of AD patients exhibiting either T2D or impaired fasting glucose (15).

Considering the close correlation between T2D and AD, it has been generally accepted that an accurate and early diagnosis of T2D, a provision of durable glycemic control and, at least in the initial phases of the disease, a convenient prevention of chronic complications (e.g., by the adoption of an healthy lifestyle, body weight control, or moderate physical exercise) may be crucial to successfully fight against these epidemics. However, as disease progresses, some pharmacological approach (including combined therapy) may be required to successfully deal with T2D and its complications. Therefore, besides more and more efficient (and with less secondary effects) anti-T2D drugs, it is of the outmost importance to develop efficient therapies that also minimize any further damage to the already injured organs, ultimately delaying or avoiding the development of long-term complications (as AD).

Herewith, we will focus primarily on the impact of T2D in CNS, particularly in the most common form of dementia, AD, as well as on some of the currently used anti-T2D drugs. More specifically, we will briefly overview the pros and cons of the promising therapeutic potential of a new class of anti-T2D drugs – the incretin/glucagon-like peptide-1 (GLP-1) mimetics – with a particular relevance to the role of exendin-4 (Exe-4) on T2D-associated neurodegeneration.

### T2D and AD: More than unrelated pathologies

As previously mentioned, impaired insulin signaling has been critically involved in the development and progression of both T2D and AD. However, other abnormalities common to both pathologies include glucose dysmetabolism, mitochondrial dysfunction, oxidative stress, or deposition of amyloidogenic proteins (2).

Under physiological conditions, glucose metabolism is critical for proper brain function (and its neuronal connections). As neurons are unable to store and synthesize glucose, this is transported across the blood–brain barrier (BBB) via glucose transporters (GLUTs), with GLUT-1, GLUT-3, and GLUT-4 constituting the most abundant isoforms (16). However, under chronic glucose dysmetabolism (as in T2D), brain damaging effects may arise, with the formation and accumulation of advanced glycation endproducts (AGEs) constituting one of the most deleterious ones (17). AGEs are formed by a sequence of events originally identified as the end products of the Maillard reaction, during which reducing sugars (e.g., glucose and fructose) react with amino groups from proteins that become auto-oxidized and form cross-linked complexes and unstable compounds (13). Besides their massive formation in diabetic patients, AGEs were also found in retinal vessels, peripheral nerves, kidneys, and CNS of aged patients (18). Moreover, the extent of amyloid-β peptide (Aβ) glycation by AGEs has been correlated with its aggregation into senile plaques, as well as with tau protein hyperphosphorylation and the subsequent formation of neurofibrillary tangles (NFTs) (2), ultimately leading to the abnormal accumulation of both AD neuropathological hallmarks (13). Strikingly, AGEs may also react with free radicals, promoting oxidative damage and further exacerbating cellular injury (11). Therefore, as T2D exacerbates the production of such deleterious molecules, it is not surprising that AGEs production (and eventually the vicious cycle of oxidative stress) may constitute another putative biochemical link between T2D and increased risk of AD.

Regarding oxidative stress, mitochondria constitute one of the major sources and targets of reactive oxygen species (ROS), and have been increasingly demonstrated to have a pivotal role in AD and diabetes pathogenesis (19). These organelles are primarily responsible for several crucial cellular processes, being also the main coordinators of energy metabolism (by generating over 90% of cellular ATP) (2). Conversely, given the mitochondria’s high susceptibility to oxidative stress-mediated injury together with the neurons’ extreme sensitivity to alterations in their mitochondrial pool, it is acceptable that the organelles’ functional impairment can be strictly correlated with AD and diabetes (5). Recently, our group demonstrated that brain mitochondria from both the 3xTgAD and chronically sucrose-treated mouse (models for AD and T2D, respectively) had similarly impaired respiratory chain and phosphorylative system that, along with an oxidative dysfunction, might account for their similar behavioral and cognitive anomalies (e.g., augmented fear and anxiety and decreased learning and memory) (20). Together with the increased brain cortical and hippocampal Aβ levels observed in sucrose-treated mice, these reports suggested that metabolic alterations usually associated with T2D could contribute for the development of AD features (20, 21), in agreement with the previously reported profound mitochondrial anomalies and increased Aβ and phosphorylated tau protein levels in brain cortex and hippocampus upon CNS-induced insulin resistance (22), as well as with the higher predisposition of T2D patients for cognitive decline and eventually AD (23).

### Insulin/insulin receptor signaling: The missing link between T2D and AD?

Insulin was traditionally recognized for the regulation of peripheral glucose metabolism (such as in adipose, liver, or muscle tissues); however, its high levels in brain were also correlated with a crucial role in CNS (24), particularly in cognition, memory, learning, and synaptic plasticity, most probably involving the complex insulin/insulin receptor (IR) signaling pathways (25). The majority of insulin in brain derives from pancreatic β-cells, being transported mostly across the BBB (26). However, some peripherally produced insulin may also directly diffuse into the CNS through the area postrema, a circumventricular region with a “leaky” BBB (27), and even more strikingly, other CNS insulin molecules may be locally synthesized and exocytotically released by pyramidal neurons (e.g., from hippocampus, prefrontal cortex, and olfactory bulb, but not glial cells) (14). Brain insulin binds rapidly to the highly abundant and ubiquitously distributed IR in the CNS (26, 28).

Downregulation of insulin uptake in T2D brain may deprive this organ from the hormone’s beneficial effects (24). Indeed, an acute increase in peripheral insulin levels may increase insulin in cerebrospinal fluid, whereas chronic peripheral hyperinsulinemia (as in insulin resistance or T2D) may downregulate IRs at BBB, impair brain insulin uptake (26), and culminate in learning, memory, and cognition deficits (28–30). These evidences further support the idea that impaired insulin function and signaling may constitute another common mechanistic link between diabetes (particularly T2D) and AD (31). This hypothesis was further reinforced by the role of insulin in the regulation of the AD neuropathological hallmarks, Aβ and tau. In this regard, insulin/IR dysfunction has been related with neuronal tau protein hyperphosphorylation and inhibition of Aβ precursor protein (AβPP) processing, thereby detaining Aβ accumulation (26). This, together with the impairment in the extracellular release of Aβ for further clearance (as in normal conditions) may further exacerbate the deleterious intraneuronal accumulation of Aβ (26). On the other hand, under an excessive amount of insulin (as in hyperinsulinemia), the hormone competes with Aβ for insulin-degrading enzyme (IDE, a metalloprotease that degrades both insulin and Aβ), allowing for the peptide accumulation and formation of senile plaques (32). However, Ho et al. reported that both diet-induced insulin resistance and a hyperinsulinemic state in an AD-like model was associated with lower IDE levels and Aβ accumulation (33), whereas Pedersen et al. showed that rosiglitazone administration (an insulin sensitizer) improved learning and memory and simultaneously reduced Aβ_1–42_ levels (the main pathological form of Aβ in brain tissues) (34). These results suggested that rosiglitazone-mediated stimulation of insulin signaling could lead to a decrease of the hormone available to compete with IDE, therefore promoting Aβ degradation (34). Further evidences on an insulin-mediated neuroprotection were previously shown by our group, with insulin decreasing both apoptotic and necrotic neuronal death upon oxidative stress, via the activation of IR/IGF-1 receptor (IGF-1R) and subsequent inhibition of glycogen synthase kinase-3β (GSK-3β) (35). This was accompanied by a protection against neuronal lipid and protein oxidation, particularly the increase in the formation of 4-hydroxynonenal (4-HNE, a byproduct of oxidation) adducts on neuronal GLUT-3 transporters upon oxidative stress (35). As a result, insulin was able to stimulate neuronal glucose uptake and its metabolization into pyruvate (in a PI3K- and ERK1/2-mediated signaling), restoring intraneuronal energy levels (36). Accordingly, we also described an insulin-related modulation of the transport of the amino acid neurotransmitters GABA and glutamate, both in T2D and/or oxidative stress (37, 38).

Insulin-like growth factors-1 (IGF-1) and -2 (IGF-2) and relaxin were shown to share structural similarities with insulin, all belonging to the same protein family (39). Similar to insulin, IGF-1 crosses the BBB, being ubiquitously distributed in rodent and human brains, and widely affecting CNS (9, 40). Hence, insulin/IR- and IGF-1/IGF-1R-mediated signaling seem to play a key role in the regulation of brain glucose metabolism, neuronal growth and differentiation, neuromodulation, synaptic transmission, memory/learning, and neuroprotection (14). For instance, *in vivo* T2D-associated impaired insulin and IGF-1 signaling was accompanied by neuronal loss, neurite degeneration, AβPP dysmetabolism and tau protein hyperphosphorylation (41, 42). Moreover, insulin may protect against neuronal apoptosis via activation of mitogen-activated protein kinase (MAPK) signaling (namely p38 MAPK) and suppression of caspase-3 activity, a pathway that may also play a role in memory and learning (25).

### Can anti-T2D therapies be potential anti-AD therapies?

It seems unquestionable that T2D and AD are two intrinsically related pathologies sharing several common mechanisms. Therefore, it has been hypothesized that a treatment directed against T2D may be beneficial in AD. Several groups worldwide have been analyzing anti-T2D drugs (either in clinical use or under clinical trials) in the context of AD. This might be facilitated by the wide range of anti-T2D compounds established, accepted, and being used worldwide; however, there are probably many more under development or awaiting approval [we must remind that T2D is a highly concerning, modern epidemic (43)].

#### “Modern” anti-T2D treatment: reaching euglycemia is essential, but not sufficient

The maintenance of glycemic control within the normal range constitutes an efficient first approach to reduce the risk of T2D-associated long-term vascular and cardiovascular complications. But, as disease progresses, its successful management may also include the control of blood pressure and lipid levels (44). Therefore, although the achievement of an optimal glycemic control remains the main goal of diabetes therapeutic management, lately this may not be enough to reduce cardiovascular risk (45).

Some of the currently used anti-diabetics include oral [e.g., biguanides, sulfonylureas (SUs), thiazolidinediones (TZDs), and dipeptidyl peptidase-IV (DPP-IV) inhibitors] (46) and injectable agents (e.g., insulin and GLP-1 analogs) (44). Other recent therapies (either under development or awaiting validation) include glucokinase activators, amylin analogs, D2-dopamine agonists, bile acid chelators, and sodium/glucose-linked transporter-2 (SGLT-2) inhibitors (47).

Based on the outcomes of several trials, e.g., Action in Diabetes and Vascular disease: preterAx and diamicroNmr Controlled Evaluation (ADVANCE), action to control cardiovascular risk in diabetes (ACCORD), and veterans affairs diabetes trial (VADT), recent guidelines establish that therapeutic achievement of euglycemia should imply a patient-adjusted prescription that considers specific patient/disease factors (47). Among the several risk factors for T2D, obesity has been one of the closest related with the disease progression and development of late complications. As such, patients’ elevated body weight should be considered when prescribing an anti-T2D treatment (48). This is even more relevant as body weight gain is a frequent secondary effect of some anti-T2D therapies (48). As such, the development of several recent drugs had into account their effects on adipogenesis and fat mass regulation (49).

Despite the controversy on the ideal levels of glycated hemoglobin A_1C_ (HbA_1C_), it is more or less consensual that T2D treatment should aim at lowering its levels (50). HbA_1C_ is a form of glycated hemoglobin that indirectly estimates the average plasma glucose levels in the last 2–3 months, giving an idea on the “long-term” (rather than “acute”) control of blood glucose levels (51). According to the United Kingdom Prospective Diabetes Study (UKPDS) and diabetes control and complications trial (DCCT), lowering HbA_1C_ levels by 1.0% reduces microvascular complications by ~30%, and the risk for myocardial infarction and death after 10 years of intensive glucose control in patients with newly diagnosed T2D (52). Therefore, the starting HbA_1C_ value is important when choosing a treatment for each T2D patient, given that most oral anti-hyperglycemic agents can reduce its values by 1.5–2.0% from baseline levels of 8.5–9.5% (53). Thus, a patient with a baseline HbA_1C_ level higher than 9.0% will probably not achieve the therapeutic goal of <7.0%, suggesting that these patients may require a combination therapy in a near future (45). Another important therapeutic target in T2D patients should be a reduction in insulin resistance (e.g., with diet, exercise, and/or drug therapy) and/or a stimulation in insulin secretion (54). However, in the later stages of disease, when its control remains inefficient, injections of exogenous insulin may be also needed (3), with the inconvenience of, e.g., repeated insulin-induced hypoglycemic episodes. In this regard, several novel agents are being developed to better address T2D pathogenesis with a minimum risk for hypoglycemia and/or other associated risk factors (as obesity).

#### Anti-T2D drugs with a potential against AD

##### Biguanides

Metformin, derived from extracts of the French Lilac (55), is the best known biguanide and remains the anti-T2D drug of choice to initiate the pharmacological approach. It is highly efficient in improving glycemic control, is not expensive (45) and has a low risk for hypoglycemia (56). Being an insulin sensitizer, metformin does not stimulate insulin secretion directly; instead, it reduces insulin-mediated hepatic glucose production (57) and increases peripheral glucose disposal, most likely as an indirect consequence of reduced glucotoxicity (47, 58). Regarding its effects on body weight, some controversy exists, with some authors describing that metformin did not affect body weight (56), whereas others reported a metformin-induced weight loss (49). This drug can be used at all stages of T2D progression, either as monotherapy or in combination with SUs and other secretagogues (e.g., meglitinides), TZDs, and insulin (47, 58). Metformin’s adverse effects include gastrointestinal distress (e.g., abdominal pain, nausea, and diarrhea in up to 50% of patients), hepatic dysfunction, congestive heart failure, dehydration, and alcoholism (58) and, thus, must be avoided by patients with increased risk for lactic acidosis (such as those with renal dysfunction) and in elderly ones (45).

Intracellularly, metformin has been shown to inhibit the mitochondrial respiratory chain complex I, thus rising AMP/ATP ratio, with the subsequent AMP binding to (and activation of) the serine/threonine kinase AMP-activated protein kinase (AMPK), an important fuel sensor and regulator (49, 59). Dimethylbiguanide inhibits cell respiration via an indirect effect targeted on the respiratory chain complex I (60–62). This is of the outmost importance, as AMPK is able to sense inappropriate AMP/ATP ratio (insufficient energetic reserves), being turned on after an increased ATP demand or in response to metabolic stresses (e.g., ischemia, hypoxia, hypoglycemia, or increased intracellular calcium levels). This kinase is also modulated by hormones and cytokines in order to restore cellular energy homeostasis (63). Importantly, such AMPK activation requires the phosphorylation of threonine-172 by the LKB1/STRAD/MO25 complex and the calmodulin-dependent protein kinase kinase-β (CAMKKβ) (63). And when this occurs, catabolic pathways (as fatty acid oxidation or glucose transport) that generate ATP are induced, whereas ATP-consuming, anabolic processes (namely fatty acid and protein synthesis) are switched off (49). Additionally, AMPK activation has been suggested to indirectly improve hepatic insulin sensitivity (47, 49), to promote GLUT-4 protein expression and glucose uptake in human adipocytes (64) and to regulate adipogenic differentiation via inhibition of peroxisome proliferator-activated receptor gamma (PPAR-γ) (49). In parallel, metformin was also described to inhibit adenylate cyclase (AC) via accumulation of AMP and related nucleotides, thus reducing cyclic adenosine monophosphate (cAMP) levels and protein kinase A (PKA) activity in mouse hepatocytes, culminating in ablation of glucagon-dependent glucose production and output (49).

**Figure 1:**
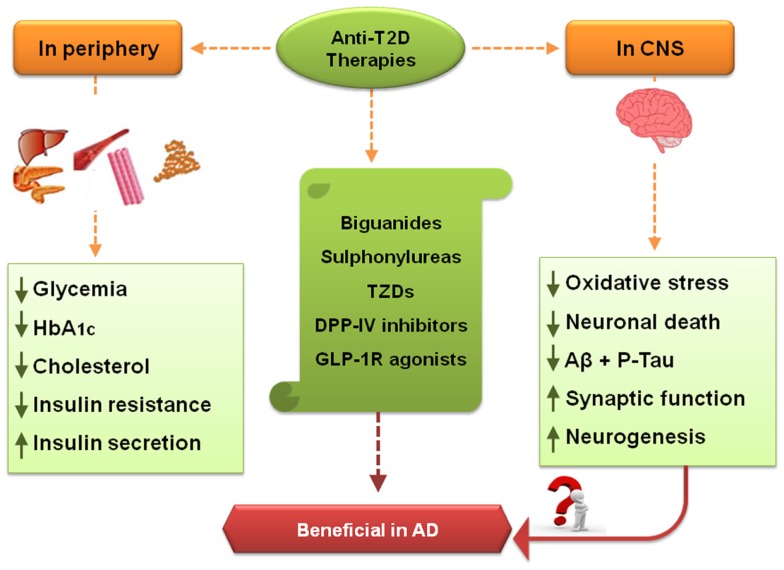
**Effects of some anti-T2D therapies – crosstalk between peripheral system and CNS**. Some of the treatments directed against T2D include biguanides, sulfonylureas (SUs), thiazolidinediones (TZDs), and the more recent incretin-based therapies, dipeptidyl peptidase-IV (DPP-IV) inhibitors and glucagon-like peptide-1 receptor (GLP-1R) agonists. In T2D patients, a few treatment goals include decrease of hyperglycemia (a typical feature of T2D), glycated hemoglobin (HbA_1C_), and total cholesterol levels. Another objective is the decrement in insulin resistance and increased endogenous insulin secretion, as in both T2D and AD insulin signaling pathways may be impaired. Some anti-T2D therapies may also positively affect CNS, namely via counteracting oxidative stress, mitochondria dysfunction, and neuronal death, together with the stimulation of neurogenesis and synaptic function. These therapies also decreased amyloid β (Aβ) accumulation and hyperphosphorylated tau protein, suggesting that they may be beneficial against AD.

Regarding the role(s) of metformin in CNS, our group showed that metformin protects T2D rat brain against oxidative stress [e.g., thiobarbituric acid reactive substances (Figure 1) and malondialdehyde levels], probably by inhibiting the antioxidant enzymes glutathione peroxidase (GPx) and glutathione reductase (GRed), by improving the levels of the non-enzymatic antioxidant glutathione (GSH) and by stimulating manganese superoxide dismutase (MnSOD) activity (65). Additionally, Guigas et al. (66) observed that metformin protected primary cortical neurons against apoptotic death. These observations, together with: (1) the inhibition of AMPK upon insulin resistance and the subsequent deterioration of energy supply, development of neuroinflammatory processes, and AD-related neurodegeneration; (2) the lower AMPK activity upon aging that may also contribute for the lower mitochondrial biogenesis and function reported in AD; (3) the protection against oxidative stress, impaired glucose transport and mitochondrial function, as well as Aβ accumulation in AD; and (4) the widely described involvement of dysfunctional brain energy metabolism (e.g., insulin resistance, impaired glucose uptake, mitochondrial and cholesterol metabolism dysfunctions, disturbed calcium homeostasis, and oxidative stress) in cognitive dysfunction and AD, led to the recent hypothesis that AMPK activation could represent a novel potential target for the treatment of neurodegenerative diseases, including AD (26, 63, 67). In line with this, the roles of metformin on the reduction of insulin resistance (Figure 1), plasma fasting insulin levels, glucose, lipid and protein synthesis, and cell growth, also stimulating fatty acid oxidation (68–70), (most of them common features to T2D and AD and involving LKB1-induced AMPK activation) rendered this drug a promising therapeutic approach also against AD (Figure 1) (62, 63). This hypothesis is further supported by the recent observations that metformin prevented the accumulation of AD-like molecular and pathological features in a differentiated neuronal cell line submitted to chronic hyperinsulinemia, including Aβ generation and tau protein hyperphosphorylation (71, 72). Although the precise underlying mechanisms are still under investigation, it is possible that such protection may occur via the activated AMPK-associated regulation of amyloid precursor protein (APP) processing and tau protein phosphorylation (AMPK is a physiological tau protein kinase) (63). Alternatively, metformin-induced AMPK activation may further inhibit the downstream mTOR signaling, thus promoting autophagy and lysosomal degradation of Aβ (63). Moreover, Kickstein et al. (62) proposed that by increasing the phosphorylation of mTOR and its downstream targets p70S6 kinase, S6, and tau proteins in primary cultured neurons, metformin and its derivative phenformin could then activate PP2A, thereby efficiently dephosphorylating tau protein. Interestingly, such effect of metformin on PP2A activity could also overcome a reduction in GSK-3β phosphorylation at serine 9 (and its subsequent activation), thus helping in the attenuation of neuronal tau protein phosphorylation, via an AMPK-independent pathway (62).

However, such promising anti-AD potential afforded by metformin, must be faced with caution, as more research is needed to clarify some of the controversial observations reported, as well as the underlying molecular mechanisms. Indeed, Chen et al. suggested that metformin could exacerbate AD pathology, as it promoted both intra and extracellular production of Aβ, probably via the AMPK-dependent transcriptional upregulation of BACE1 (73). Moreover, Li et al. (71) reported recently that, despite the amelioration in c-Jun-N-terminal kinase activity (JNK, a kinase known to increase tau protein phosphorylation) and in hippocampal expression of the synaptic protein synaptophysin in the metformin-treated *db/db* mouse model of T2D, this was not accompanied by an amelioration of spatial learning and memory impairments. Even other authors reported that AMPK activation could be detrimental, e.g., in stroke and Huntington disease (74–77). Our group also reported that metformin could exacerbate rat liver cell death via induction of hepatic mitochondrial dysfunction (78), in accordance with the widely described hepatic dysfunction associated with metformin (79). Therefore, it seems that depending on the conditions and biochemical settings, AMPK (and ultimately metformin) might be a two-edged sword regarding Aβ production and tau protein phosphorylation, and ultimately AD pathology (63). In this perspective, we believe that it will be of outmost importance to unravel if AMPK activation plays a causative or a protective role, if this is a consequence of AD and if this is clinically beneficial in humans (63), e.g., concerning metformin or other AMPK-stimulating compounds.

##### Sulfonylureas

As previously referred, SUs are used as monotherapy or combined with other oral agent classes and insulin (46). Second-generation SUs (glyburide, glipizide, and glimepiride) are more potent and probably safer than the first-generation ones (chlorpropamide, tolbutamide, acetohexamide, and tolazamide), but have a similar efficacy (46). These drugs are the second leading choice (after the biguanide metformin) in anti-hyperglycemic agents worldwide, most likely because they are rather inexpensive and quite efficient (47). Although SUs treatment aims essentially at lowering blood glucose and to allow for insulin release at a lower glucose threshold than normal, we must bear in mind that these drugs also appear to increase the risk of hypoglycemia (particularly in elderly patients with renal dysfunction, and/or with irregular meal schedules) (46, 47). Moreover, a clinical trial showed that SUs were able to decrease HbA_1C_ up to 7.4% (80), but it has been more commonly reported only a modest 1–2% lowering (81). Others described that SUs reduced hypercoagulation and cardiovascular damage, and modestly protected pancreatic islet cells against the progressive cellular deterioration upon T2D (45). However, in an early clinical trial, Kilo et al. (82) observed that although SUs-treated patients had an increased cardiovascular mortality, overall there were no significant differences between SU-treated subjects and those treated with insulin. Importantly, another highly relevant issue in SUs therapy is weight gain (typically from 2 to 5 kg), which might be of the outmost importance in the context of the above-mentioned close relation between diabetes and obesity (58). Finally, as most of these drugs are metabolized in liver and cleared by the kidney, some cautious is needed regarding patients with advanced forms of either hepatic or renal disability (46).

Despite the limited knowledge on the precise molecular mechanisms involved in SUs-induced insulin secretion, it has been suggested that, after their binding to the SU receptors at the surface of pancreatic β-cells, ATP-sensitive potassium (K_ATP_) channels are closed, cell membrane depolarization and calcium influx occur, ultimately leading to the glucose-independent insulin secretion (46, 47) (Figure 1). Alternatively, others suggested that SUs may have PPAR-γ agonist activity (49). Also, unclear is the SUs-induced stimulation of insulin action in adipocytes, with some data pointing toward an SU receptor-independent mechanism, as the receptors are not expressed in adipose tissues (83). Regarding the eventual SUs’s potential therapeutic action against dementia and AD, evidences from literature are scarce. In a recent study, Exalto et al. (84) reported that the occurrence of dementia in SUs-treated T2D patients was decreased and, although a correlation between the specific actions of the drug *per se* and improved glycemic control could be involved herein (85) (Figure 1), the precise mechanisms remain unknown. Thus, further investigation to clarify the molecular mechanisms of action of SUs (particularly in CNS and in AD-dementia type) is needed before assuming their anti-AD potentialities.

##### Thiazolidinediones

The members of this class of drugs (also named glitazones and currently represented by rosiglitazone and pioglitazone) act as agonists of PPAR-γ, a nuclear receptor highly expressed in insulin-sensitive tissues (e.g., adipocytes, liver, and pancreas) that regulates the transcription of genes involved in lipid and glucose metabolism (45, 49). Once activated, PPAR-γ stimulates adipocyte differentiation (86) and decreases hepatic glucose production (87). Additionally, in placebo-controlled trials, TZDs were as efficient as SUs and metformin in decreasing HbA_1C_ levels, but more efficient than α-glucosidase inhibitors (46). Moreover, TZDs were more efficient in maintaining long-term cell function and glycemic control than metformin (45), and to decrease insulin resistance in peripheral tissues (88) (Figure 1), with data suggesting that TZDs may actually prolong β-cell survival (89). However, TZDs have been increasingly associated with a few problematic side effects: (1) relatively expensive, (2) weight gain, (3) fluid retention, and (4) increased myocardial infarction risk, particularly by the use of rosiglitazone (which has been restricted in the United States and even suspended in the European Union). On the other hand, although pioglitazone appeared to reduce major cardiovascular events and death (90), its use has been also linked with an increased risk to fractures and bladder cancer (47).

Regarding the intracellular effects of TZDs, it is known that their action is not to stimulate insulin secretion by pancreatic β-cells directly (in fact, its local concentrations usually remain low); rather, TZDs aim at enhancing β-cells’ sensitivity and efficiency (91), most likely by decreasing circulating glucose and free fatty acid levels (both having deleterious effects on insulin secretion), through their collection in adipose tissue (92). Although the molecular mechanisms underlying the insulin-stimulated glucose uptake (e.g., by skeletal muscle cells) upon TZDs treatment (one of their most prominent effect) remain mostly unclear, it has been proposed that this might largely occur indirectly via the interaction between TZDs and adipocytes (49). The resulting upregulation of GLUT-1 and GLUT-4 would then improve glucose disposal and consequently reduce its toxicity (49). Despite the lack of knowledge on the intermediary signal(s) between fat and muscle, it is possible that leptin, free fatty acids, tumor necrosis factor α (TNFα), adiponectin, and the more recently isolated resistin could play a role (46).

Similarly to SUs, evidence on the anti-AD potential of TZDs is scarce and, in our perspective, this might be due to not only the lack of information on the precise molecular mechanisms underlying their effect(s) (particularly in brain) but also the limitations currently posed to their therapeutic use in T2D patients worldwide due to their potentially serious side effects. Nevertheless, in 2005, Dormandy et al. reported that TZDs downregulated *in vitro* Aβ deposition, whereas Neumann et al. (93) observed that troglitazone reduces tau protein phosphorylation at serines 202 and 396/404 (known to be phosphorylated in early and later stages in AD, respectively, as well as in other neurodegenerative pathologies) (Figure 1). This, together with the rosiglitazone-induced preservation of memory function and selective attention and the decrement in plasma levels of Aβ_1–40_ and Aβ_1–42_ in AD patients AD (94, 95). However, this hypothesis deserves further investigation and should be faced carefully given the adverse side effects of TZDs.

##### Insulin

Insulin is the best known anti-type 1 diabetic drug, being a life-saving treatment in these patients. Regarding T2D individuals, although insulin has been increasingly used to control their blood glucose levels and prevent chronic complications, its efficiency still remains controversial (47, 96). Currently, several different human insulin formulations and insulin analogs are available commercially, aiming at “personalizing,” as closely as possible, the normal insulin physiology (47) and thereby minimizing its high risk for hypoglycemia (45).

Besides the above-mentioned relevance of insulin in both T2D and AD brains (particularly endogenous insulin and/or its downstream signaling cascades), in terms of exogenously administered hormone, it is of the outmost importance to refer that insulin-induced hypoglycemia has been long associated with neurological and cognitive deficits, seizures, coma, and neuronal death (97), particularly in hippocampus, external cortical layers and striatum (98). Although the mechanisms involved herein remain poorly understood, our group has recently demonstrated that insulin-induced hypoglycemia impairs brain mitochondria function and increases oxidative stress (99, 100), leading to a parallel increase in plasma and/or brain cortical aspartate, glutamate, glutamine, and taurine, and a lowering in GABA levels in rats submitted to an acute episode of insulin-induced hypoglycemia (96). This was accompanied by a further release of aspartate, glutamate, and taurine from depolarized hypoglycemic synaptosomes, suggesting that an additional metabolic insult may be sufficient to exacerbate the release of excitatory aminoacids. Therefore, this may constitute an additional mechanism underlying diabetes (and, more particularly, insulin-induced hypoglycemia)-associated neuronal injury, degeneration, and cognitive impairment (96). In this perspective, besides these adverse effects, we must be aware that exogenously added insulin has been demonstrated to play several beneficial roles in CNS, with the ubiquitous localization of IRs in hippocampus, entorhinal, and frontal cortices further pointing toward the hormone’s benefits in brain. Moreover, the primary location of IRs at the synapses, where their signaling may contribute to synaptic remodeling (together with a role on memory formation under normal metabolic conditions) (101), and the long known modulatory role for insulin against deleterious Aβ synaptic accumulation and effects (42), further suggest that insulin administration (without hypoglycemic episodes) or, even better, the pharmacologic restoration of brain insulin sensitivity and action could be one of the best promising therapeutic approaches against AD.

##### Incretins/GLP-1 receptor agonists and DPP-IV inhibitors: the “dream team”?

From the above, one of the most efficient therapeutic approaches to T2D (and apparently also against neurodegeneration and AD) are the novel classes of GLP-1 analogs and DPP-IV inhibitors. Incretins are gastrointestinal hormones, first identified in the 1960s, when a glucose load was observed to increase insulin secretion (102). This was due to the secretion of both GLP-1 (an incretin of 30 amino acid length) and glucose-dependent insulinotropic polypeptide [also called gastric inhibitory polypeptide (GIP)], by enteroendocrine K and L cells, respectively (44, 103). Recently, it was described that, together, both hormones account for the majority of glucose-dependent insulin production, secretion, and response (1, 103). Indeed, the earliest endocrine metabolism activated after a meal is the insulinotropic incretin effect that consists on the activation of the potent insulinotropic GLP-1 immediately after GIP secretion (104). Although the precise mechanisms involved herein remain somehow controversial, it is plausible that they may involve both central and peripheral effects of GLP-1 to promote insulin and inhibit glucagon secretion (thus dealing with the post-prandial rise in glycemia and the maintenance of blood glucose homeostasis) (1, 103, 104), reduce gastric emptying, appetite, food intake, and body weight (47, 103, 105–107).

**Figure 2:**
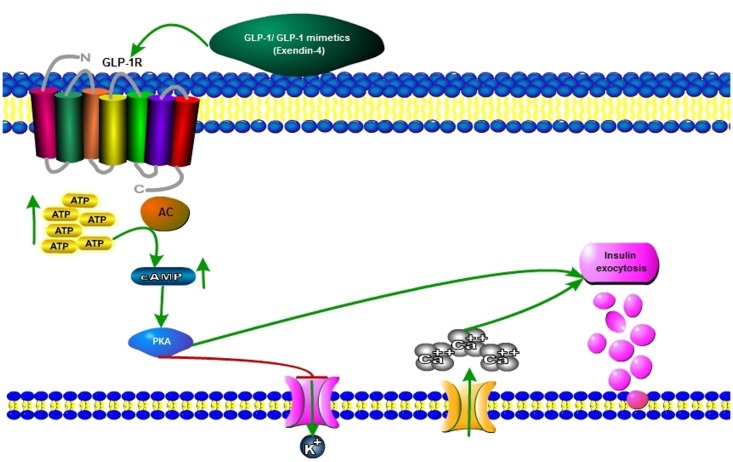
**Mechanisms of first phase of insulin secretion by GLP-1 mimetics**. GLP-1 and GLP-1R agonists exert their actions by binding to GLP-1R, a G-protein-coupled receptor (GPCR). GLP-1R is ubiquitously expressed throughout the whole body, including peripheral and central nervous systems. Despite some controversy, it has been hypothesized that incretin receptor activation in β-cells after a meal leads to glucose metabolization through glycolysis and the subsequent increase in cytosolic ATP content. Then, the hyperpolarizing K_ATP_ channels close and β-cell membrane depolarizes, allowing calcium influx to occur via the voltage-dependent calcium channels (VDCC) and culminating in calcium-dependent insulin exocytosis. Alternatively, insulin secretion may be also stimulated by intracellular signaling cascades involving, e.g., cyclic AMP (cAMP) or its downstream targets Epac (exchange protein activated by cAMP), protein kinase A (PKA), AMP kinase (AMPK), protein kinase C (PKC), or MAPK. As blood glucose levels return to normal, GLP-1-induced insulin exocytosis is decreased.

Molecularly, GIP and GLP-1 bind and activate structurally distinct G-protein-coupled receptors (GPCRs) (27), with GIP receptor being predominantly expressed on islet β-cells and (to a lesser extent) in adipose tissue and in CNS, whereas GLP-1 receptor (GLP-1R) occurs in islet α and β cells, as well as in central and peripheral nervous systems, heart, kidney, lung, and gastrointestinal tract (27). Under physiological conditions, insulin secretion may depend on the GLP-1 receptor (GLP-1R)-mediated aerobic metabolization of glucose through glycolysis and the subsequent raising in cytosolic ATP levels (108). As a consequence, the hyperpolarizing K_ATP_ channels close, β-cell membrane depolarization occurs, allowing calcium influx through voltage-dependent calcium channels (VDCC) occurs and calcium-dependent insulin exocytosis is potentiated (105, 106, 108) (Figure 2). Alternatively, insulin secretion may be stimulated by intracellular signaling cascades involving, e.g., cAMP or its downstream target Epac (exchange protein activated by cAMP), PKA, AMPK, protein kinase C (PKC), and MAPK, as it has been described for GLP-1-mediated stimulation of glucose-dependent insulin release (108). As blood glucose levels return to normal, GLP-1-induced insulin exocytosis is decreased (45, 103). Despite all this, the effects of incretins on pancreatic β-cell intermediary metabolism to promote insulin secretion still remain controversial. Tsuboi et al. reported that GLP-1 is able to modulate β-cell intermediary metabolism (and function) at both low and high glucose levels, probably by changing cytosolic ATP content in a GLP-1R-dependent manner (109). Conversely, others failed to observe any effect of exendin-4 (a GLP-1R agonist) on mitochondrial ATP levels in primary rodent islets (110), despite the large oscillations in intracellular calcium, and the subsequent activation of ATP-consuming and – production processes (and, thereby, of cellular metabolism) to maintain intracellular calcium homeostasis (108). Alternatively, Burmeister et al. (106) proposed recently that GLP-1R-associated increase in glucose metabolism after a meal could initiate a feedback inhibition of food intake, mediated by a decrement in AMPK activity. As such, glucose could be considered as both a stimulus and a mechanistic component of GLP-1-mediated suppression of food intake (106).

Circulating human GLP-1 has a short half-life (1–2 min), being almost completely degraded by DPP-IV (111), an ubiquitously expressed aminopeptidase that occurs, e.g., in liver, lung, kidney, endothelium, and lymphocytes, and is essential for incretin inactivation (112). This renders the therapeutic use of the naturally occurring human GLP-1 highly unfeasible; thus, several long-acting GLP-1R agonists that mimic the effects of endogenous GLP-1 and more resistant to DPP-IV-mediated degradation have been developed as anti-T2D therapies (113). In line with this, mice with targeted downregulation of DPP-IV showed higher plasma levels of GIP and GLP-1, increased insulin secretion, and reduced glucose release (114), whereas DPP-IV inhibitors were reported to reduce HbA_1C_ levels by 0.7% in T2D patients undergoing metformin therapy (115) (Figure 1). Besides incretins, other DPP-IV substrates include other gastrointestinal hormones, neuropeptides [e.g., neuropeptide Y (NPY)], cytokines, and chemokines (103) that may also affect lipid metabolism and adipogenesis (49). Indeed, blockade of the NPY receptor was reported to inhibit the DPP-IV-mediated stimulation of lipid accumulation (49). Similar to GLP-1 analogs, DPP-IV inhibitors often become less effective as insulin resistance progresses and pancreatic β-cells function deteriorates (113). In general, DPP-IV inhibitors are well tolerated, can be given orally to lower fasting and post-prandial glucose, apparently without affecting gastric emptying or body weight (45, 49). These drugs can be used either as monotherapy or together with other oral agents or insulin. Amongst DPP-IV inhibitors, sitagliptin, saxagliptin, and linagliptin have been approved by the FDA, whereas vildagliptin is available in the European Union and alogliptin in Japan (47). At their recommended doses, a daily administration of these DPP-IV inhibitors prevented DPP-IV activity for at least 24 h, with the exception of vildagliptin that required a twice daily administration (116). Importantly, as ~80% of non-metabolized sitagliptin, alogliptin, and saxagliptin were detected in urine (renal excretion is their main excretory pathway), DPP-IV inhibitors may require an additional dose adjustment in patients with impaired renal function (116). Regarding vildagliptin, it is metabolized mostly by the liver, not being recommended to patients with hepatic insufficiency (47), whereas linagliptin is only minimally metabolized and, thus, 78% of the administered dose is excreted unchanged by the hepatobiliary route via the feces, being other 5% excreted through the kidney (116). In this perspective, linagliptine could be an attractive therapeutic option for T2D patients with moderate, severe, or end stage renal disease; nevertheless, it should be prescribed cautiously to patients with hepatic insufficiency (47).

Importantly herein, recent studies showed that sitagliptin delayed the development of AD neuropathological hallmarks in an adult, double transgenic mouse model of AD (117), or even improved learning behavior in adult, insulin-resistant rats (117, 118). More specifically, chronic administration of sitagliptin reduced both hippocampal APP and Aβ deposition in the transgenic AD mice (117) (Figure 1), whereas in the insulin-resistant rats, it promoted a decrease in plasma insulin, cholesterol, and HDL levels, and ameliorated the HOMA values (an index of insulin resistance) (118). Additionally, both vildagliptin and sitagliptin were able to protect against oxidative stress in insulin resistance rats (118) (Figure 1). Despite these promising results of DPP-IV inhibitors’ administration against such deleterious conditions for the rodent brains, the knowledge on the precise underlying mechanisms remains scarce and will be of the outmost relevance in the context of the DPP-IV inhibitors’ potential as anti-AD drugs.

Clinical studies comparing GLP-1R agonists vs. DPP-IV inhibitors revealed that exenatide or liraglutide (GLP-1R agonists currently used in T2D treatment) were more efficient than sitagliptin (a DPP-IV inhibitor) in lowering blood glucose levels, upon hyperglycemia (Figure 1), and body weight in T2D patients (1, 119). Conversely to insulin and other oral anti-T2D, the risk of hypoglycemia was also low and comparable between GLP-1R agonists and DPP-IV inhibitors (47), thereby constituting an additional advantage for their possible application in AD treatment, as further discussed in the next section.

##### GLP-1 analogs and GLP-1R agonists: what is effective against T2D could be also effective against neurodegeneration and AD?

As previously described, a crucial peripheral effect of GLP-1 analogs and GLP-1R agonists is to promote β-cell function, since β-cell mass and function are often already impaired at the time of T2D diagnosis (1). Amongst the clinically available long-acting GLP-1 analogs for the treatment of T2D, liraglutide is the mostly used one, either as mono- or combined therapy with metformin or TZDs (47). Liraglutide shares 96% structural resemblance with human GLP-1 and besides its traditional role in lowering blood glucose levels toward euglycemia, it has been also shown to reduce systolic blood pressure, as well as to decrease the risk for hypoglycemia when combined with SUs (47). Importantly, given such interesting features of these incretin hormone’s analogs (with a special emphasis on their unique feature of promoting glucose-dependent insulin secretion with only a minimal risk of damage associated with repeated hypoglycemia episodes), intense research efforts have been done to clarify their precise molecular mechanisms of action and, simultaneously, to develop and test other similar drugs.

Given that GLP-1 is ubiquitously expressed in CNS, particularly in hypothalamus, cortex, hippocampus, striatum, substantia nigra, brain stem, and subventricular zone (an area of adult brain neurogenesis) (113), it is not surprising that not only GLP-1’s main peripheral roles might be centrally controlled but also that this incretin and its analogs may play a pivotal role in brain (Figure 1). In fact, as brain GLP-1 has been increasingly suggested to be neuroprotective, it is possible that the modulation of its specific receptor may represent a promising strategy against neurodegeneration/death and memory and cognition impairment, i.e., it may constitute a putative pharmacological target against age- and/or T2D-related neurodegenerative diseases, including AD. In line with this, During et al. observed that knockout mice for GLP-1R had memory and learning deficits (120). Additionally, the GLP-1 analog liraglutide was shown to prevent hippocampal synaptic loss and plasticity (Figure 1), and memory impairment in the APP/PS1 mouse model of AD (121). Recently, other authors reported that liraglutide hampered Aβ plaque formation (122) (Figure 1), reduced astrocyte- and microglia-mediated inflammatory responses (123), and promoted neurogenesis (Figure 1) and neuronal proliferation in dentate gyrus (124). Although these results point toward the beneficial use of liraglutide as an anti-AD pharmacological approach, further clarifying research is needed, with a particular emphasis on the ongoing clinical trials (“Identifying potential effects of liraglutide on degenerative changes” – ClinicalTrials.gov Identifier: NCT01469351; and “Evaluating liraglutide in Alzheimer’s disease” – ClinicalTrials.gov Identifier: NCT01843075).

Besides GLP-1 analogs (as liraglutide), the incretin class of anti-T2D drugs also include the GLP-1R agonists. Amongst them, the most widely clinically used and best studied is exenatide (exendin-4, Exe-4), a highly effective molecule against T2D (125). Exenatide is a synthetic injectable GLP-1R agonist resistant to DPP-IV, being derived from Exe-4 (a peptide obtained from the saliva of the Gila monster, *Heloderma suspectum*) (126). Exe-4 shares a 53% amino acid sequence identity with human GLP-1, being more effective in lowering glucose levels than the native hormone (103). Clinically, Exe-4 constitutes an important complement to diet and exercise in the improvement of glycemic control in T2D adults (1), either when used as a monotherapy or in combination with other oral anti-T2D agents (127). Additionally, Exe-4 decreases food intake (128) and, together with metformin and/or SUs, lowers HbA_1C_ levels by 0.8–0.9% (Figure 1) and reduces body weight by 1.6–2.8 kg (129). Interestingly, exenatide promoted graft survival and function after islet-cell transplantation in T1D patients (130).

Although the molecular mechanisms involved in the potent effects of Exe-4 on glucose-dependent insulin secretion (Figure 1) and insulin gene expression (131) are still under intense research, it is possible that they may involve drug binding and activation of GLP-1R in pancreatic β-cells. These receptors belong to the G-protein-coupled class of receptors, activating adenyl cyclase and rapidly increasing cAMP levels, thus promoting downstream intracellular signaling cascades (27) that may culminate in the modulation of β-cell proliferation and function, as well as in the inhibition of apoptosis (103, 128). Importantly, some patients treated with exenatide reported some transient secondary effects, including nausea, vomiting, and diarrhea (103, 132) and, more rarely, pancreatitis (133).

Given its stability in blood and high lipophilicity, most of the peripherally injected Exe-4 crosses the BBB without being trapped by endothelial cells and rapidly reaches the brain (104). Additionally, Exe-4 action is insensitive to food deprivation for 24 h (134). Besides the periphery, GLP-1R is also highly expressed in CNS (135) and, therefore, it is conceivable that the activation of downstream GLP-1R-mediated signaling molecules may at least partially be involved in Exe-4-mediated stimulation of neurogenesis in subventricular zone (136), neurite outgrowth, neuronal differentiation, rescue of degenerating neuronal cells, and protection against both *in vitro* and *in vivo* excitotoxic damage (137) (Figure 1). However, the underlying mechanisms remain poorly understood and deserve further clarification. In clinical studies, Exe-4 was shown to promote cellular neogenesis, proliferation, and apoptotic death inhibition (131). In adult rodents, it was shown to improve hippocampus-associated behavior (138) and to protect against neuroinflammation (132). Moreover, Exe-4 protected against Aβ-associated hippocampal neuronal death (Figure 1) and rescued learning and memory in intracerebroventricularly injected streptozotocin rats (a model of sporadic AD) (1). Thus, besides its high relevance and efficacy in the therapeutic management of T2D, Exe-4 is also faced as a promising drug for the treatment of dementia and AD. In this regard, we believe that the results from the ongoing clinical trial “A pilot clinical trial of exendin-4 in Alzheimer’s disease” – ClinicalTrials.goc Identifier: NCT01255163, will be of the outmost relevance herein.

Amongst the novel GLP-1R agonists, lixisenatide and albiglutide are currently undergoing phase III clinical trials. Lixisenatide is based on exendin-4 (1–39) molecule and has a modified C-terminal with six additional lysine residues that confers a fourfold higher affinity to the GLP-1R than the human GLP-1, together with a half-life of about 3 h (139, 140). Similar to liraglutide, lixisenatide also crosses the BBB into CNS, whereby it has been shown to exert strong neurogenic effects in an AD rodent model (124, 141) (Figure 1), as well as to prevent Aβ-related impaired synaptic plasticity, hippocampal LTP, and spatial learning memory in a PI3K/Akt/GSK-3β-mediated pathway (142). Despite such potent brain protective effects, further *in vivo* and clinical research aiming at the treatment of neurodegenerative diseases with lixisenatide are needed. Regarding albiglutide, this molecule consists of two GLP-1 (7–36) molecules connected to recombinant human albumin (143), in which a single amino acid substitution (ala → gly) renders it resistant to DPP-IV, with a half-life of ~5 days that allows for a weekly dosing (144). Unfortunately, its size renders albiglutide unable to cross the BBB (145), thereby rendering the potential peripheral use of albiglutide against neurodegenerative disorders somehow unfeasible and first requiring the development of efficient CNS delivery strategies. Importantly, albiglutide’s adverse effects include nausea, injection site reactions (47), and eventually some degree of gastrointestinal intolerability (145), which must be taken into account when prescribing this drug to patients with gastrointestinal problems.

## Conclusion

Type 2 diabetes patients often develop some form of dementia (such as AD), whereas AD patients may also present hyperglycemia, hypercholesterolemia, and insulin signaling dysfunction (common features to T2D). Thus, it has been increasingly suggested that several anti-T2D drugs may have a therapeutic potential in dementia, with some of them already under clinical analysis for that purpose. In fact, some of the above-mentioned anti-diabetics were beneficial against some AD hallmarks, e.g., Aβ plaque formation and tau hyperphosphorylation. Some of them also promoted neurogenesis and cell proliferation, and reduced neuroinflammation and cell death. However, despite so many promising preventive and/or treatment strategies against human dementia, a precise knowledge on (1) the underlying common mechanisms between T2D and AD, and (2) the molecular determinants of such drugs’ effects, both peripherally and centrally, with a special emphasis on T2D- and/or AD-related damage, is urgently needed to unquestionably consider that “what is good for T2D treatment is also good for AD.”

## Conflict of Interest Statement

The authors declare that the research was conducted in the absence of any commercial or financial relationships that could be construed as a potential conflict of interest.
